# Pad-Printing as a Fabrication Process for Flexible and Compact Multilayer Circuits

**DOI:** 10.3390/s21206802

**Published:** 2021-10-13

**Authors:** Ahmad Jaafar, Spyridon Schoinas, Philippe Passeraub

**Affiliations:** Institute of Industrial Sciences and Technologies, University of Applied Sciences and Arts Western Switzerland (HEPIA/HES-SO Geneva), CH-1202 Geneva, Switzerland; spyridon.schoinas@hesge.ch (S.S.); philippe.passeraub@hesge.ch (P.P.)

**Keywords:** pad-printing, conductive inks, insulating inks, UV curable inks, dielectric inks, multilayer printing, printed electronics, flexible electronics, additive manufacturing

## Abstract

The purpose of this paper is to present a newly developed process for the fabrication of multilayer circuits based on the pad-printing technique. Even though the maturity level, in terms of accuracy, substrate type and print size of several printing industrial processes is relatively high, the fabrication complexity of multilayer printed electronics remains relatively high. Due to its versatility, the pad-printing technique allows the superposition of printed conductive and insulating layers. Compared to other printing processes, its main advantage is the ability to print on various substrates even on flexible, curved or irregular surfaces. Silver-based inks were used for the formulation of conductive layers while UV inks were employed to fulfil the functionality of the insulating layers. To demonstrate the functionality of the pad-printing results, a multilayer test pattern has been designed and printed on Kapton^®^. Furthermore, to demonstrate the efficacy of this approach, a multilayer circuit composed of three stacked layers has been designed and printed on various substrates including Kapton^®^, paper and wood. This electronic circuit controls an array of LEDs through the manipulation of a two-key capacitive touch sensor. This study, allowed us to define recommendations for the different parameters leading to high printing quality. We expect a long-term beneficial impact of this study towards a low-cost, fast, and environmental-friendly production of printed electronics.

## 1. Introduction

Thanks to the advances of 2D Additive Manufacturing (2D AM) techniques, production flexibility and high-speed characteristics have been obtained, leading to high throughput processes and low-cost manufacturing platforms. The surging impact of these platforms in the manufacturing of printed electronics (PE) continues to grow [1]. Compared to traditional electronic microfabrication techniques such as photolithography and electroplating, 2D AM techniques have obtained a competitive advantage. This is not only the case in terms of rapidity and cost reduction but also in terms of eco-friendliness, as 2D AM techniques reduce used and wasted materials [2].

Digitalization trends such as the Internet of Things (IoT) and industry 4.0 created a further need for lightweight, low-cost, flexible, and eco-friendly electronics, which cannot be covered by traditional electronic fabrication methods. Current 2D AM methods allow for all these attractive characteristics that make electronics appropriate for these applications [3]. Several applications, as well as various manufacturing processes of flexible and stretchable printed electronics, are highlighted in a detailed study in [4].

2D AM methods have already been well established for applications in sensor systems [5], OLED displays, RFID tags [6], and passive electronic components such as capacitors, inductors, and resistors [7].

The 2D AM techniques most used for the fabrication of PE are inkjet printing, screen printing, offset gravure, and pad-printing. A comparison between these different printing techniques for the fabrication of solar cells was carried out in [8]. Each 2D AM method should be chosen to meet the requirements of the specific application, due to their different and peculiar characteristics. Inkjet printing is a popular process due to its ability to print without contact and its advantage of digital patterning [9]. However, this method is limited by aspects related to low throughput production [10] and nozzle clogging [7]. Primary batteries were fabricated by the use of screen-printing in [11]. Screen printing is an attractive process in terms of productivity and cost. Due to its inability to print on non-flat surfaces, the types of substrates used in screen-printing are limited. In [12], the offset lithography printing process was employed for the fabrication of capacitive-type relative humidity sensors. Despite its cost and throughput attractiveness, offset printing is not suitable for small production runs and cannot print on curved surfaces.

Pad-printing is a versatile offset printing technique that allows high-quality layer-by-layer printing. Applications that could benefit from the expansion of pad-printing include sensors, biosensors, RFID tags as well as photovoltaic cells. An assessment of the potential of pad-printing in the production of RFID antennas was presented in [13]. Their performance showed compatibility with industry standards. Similarly, a UHF RFID tag manufacturing by pad-printing on a convex surface was demonstrated in [14]. Small antennas were pad-printed onto the thermoplastic hemisphere using composite ink; they were presented in [15]. In addition, a pad-printed piezoelectric film on a concave surface was presented in [16]. An Electro-Luminescence (EL) display device has been printed on a curved surface using the pad-printing method, developed in [17]. The influence of the printing parameters was evaluated to produce EL panels [18]. The printing process of the latter EL device was presented in [19]. Pad-printed disposable electrode systems have been evaluated in [20]. A pad-printed flexible fluxgate sensor was presented in [21]. In [22], the pad-printing process is used as an additive step for the manufacture of dielectric elastomer actuators showing the simplicity and the flexibility of this process. Optimized pad-printed source-drain layer structure was presented in [23]. The realization of the fine-line sensor structure (50 µm) was also achieved using the pad-printing technique [24]. In addition, it has been shown in [25] that the pad-printing technique is a possible solution for preparing the active layer of a solar cell.

The main advantages of pad-printing, over other printing processes, are its capability of handling high-viscosity inks and its ability to print on a variety of objects including flat, spherical, cylindrical, texture, concave, or convex surfaces. Other advantages of pad-printing are its ability to be used for prototyping, or as an intermediate or additive process to other production techniques, its adaptability to substrates with different sizes, its compatibility with small to medium run production and the small amount of ink used. The main constraint of pad-printing is the size of the printed image, which is limited by the dimensions of the machine and the pad. It is also difficult to achieve thick films of more than 20 µm in pad-printing, unlike the screen-printing method.

Despite the rapid development of these 2D AM methods, the problem of multilayer printing is hardly addressed, which remains a major drawback compared to traditional electronic manufacturing techniques. Multilayer PE involve employing a printing process to print functional layers deposited on top of each other layer by layer. The functional layers could be insulators, semiconductors, or conductors. Unlike single-layer printing, the complexity increases when printing functional multilayers because each newly deposited layer depends on the quality of the printing of the layer below. Furthermore, ink components and especially thinners from each newly deposited layer deteriorate the layer below. Due to the complexity of multilayer printing, the number of industrial applications remains limited. Among studies that have shared the same interest in printing functional multilayers, we found a combination of E-jet (electrohydrodynamic inkjet) to print conductive tracks with piezo-based inkjet to print the insulating layer [26] interesting. The long printing time of each layer (between 50 s and 125 s), in combination with the minimum number of printed layers (25 layers) and the challenging process control required for the E-jet process, decrease the attractiveness of this approach. Fully inkjet-printed multilayer capacitors and inductors were fabricated in [27] expressing the need for multilayer printing optimization.

The work presented here shows a process based on the pad-printing technique that is able to overcome such limitations. By the optimization of the various parameters of the pad-printing, multilayer electronic circuits can be obtained. More specifically, this study aims to reinforce the multilayer pad-printing of functional inks in terms of attractiveness and robustness. This work also intends to demonstrate its implementation potential and to point out its associated constraints. We show the added value of pad-printing UV inks to obtain multilayer circuits. To validate the developed printing process, we used a condensator-like test device comprising three superimposed layers and a demonstrator circuit that controls an array of LEDs through a two-key capacitive touch sensor. To the best of our knowledge, no pad-printed multilayer circuit has been reported in the literature in the past. This study is novel for its utilization of resin-based UV inks as insulating layers in combination with silver-based conductive inks for the creation of multilayer circuits.

## 2. Materials and Methods

### 2.1. Materials

“SpectraZ 14” silver-based inks were used for the creation of the conductive layers. They are specially developed by the Laboratory of Microengineering and Bioinstrumentation of the Institute of Industrial Technologies and Sciences from the University of Applied Sciences and Arts Western Switzerland. Resin-based UV ink “TPC 728” (Teca-Print AG, Thayngen, Switzerland) was used for printing the insulating layers. “VS” thinner (Teca-Print AG, Thayngen, Switzerland) was used for the formulation of the UV inks. “Kapton^®^” 100FPC (DuPont, Wilmington, CT, USA) polyamide film with 125 µm thickness was used as a substrate. Wooden boards of various sizes, as well as cardboard business cards 85 mm × 54 mm, were also used as substrates. The type of pad used in this study is a special PDMS pad called “Dual” (Teca-Print AG, Thayngen, Switzerland) of conical shape with 54 Shore hardness and rectangular shape with 64 Shore hardness. Teca-Print engraved plates (clichés) (Teca-Print AG, Thayngen, Switzerland) used in this work are made of 0.5 mm thick laser engraved rectified steel with an engraving depth of 20 µm.

### 2.2. Methods

#### 2.2.1. Fabrication Method

**Figure 1:**
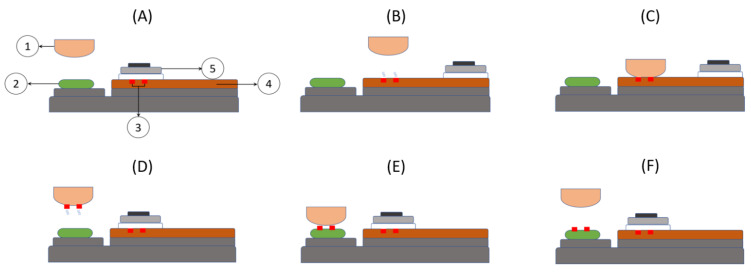
Schematic view illustrating the pad-printing process. The pad-printing cycle takes place in several steps: (**A**) Rest position with (1) Pad, (2) Substrate, (3) Inked engraved pattern, (4) Cliché and (5) Ink cup. (**B**) The ink cup moves to allow the thinner from the top surface of the inked engraved pattern to evaporate. At the same time, the pad moves laterally to the position above the ink to be transferred. (**C**) The pad moves down to pick up the patterned ink film. (**D**) The pad moves up first and then moves laterally above the substrate. During this time, the thinner from the lower surface of the ink film evaporates. (**E**) The pad moves down in contact with the substrate to deposit the ink film while the ink cup returns over the cliché pattern to fill it with ink. (**F**) The pad goes up and the ink layer is deposited on the substrate.

For the fabrication of the test devices and the demonstrator, a “TPX 301” (Teca-Print AG, Thayngen, Switzerland) pad-printing machine was used. One cycle of the pad-printing process takes place in several stages as shown in Figure 1.

Pad-printing inks are generally based on organic thinners because this process largely depends on the thermodynamic thinner evaporation process to improve the efficiency of ink transfer. Silver-based inks are obtained by introducing silver particles dispersed in a non-conductive matrix. When the volume fraction of the conductive phase begins to create continuous pathways for electronic conduction, a point called the percolation threshold is reached. Below this threshold, the ink is not conductive. An excessive increase in the volume fraction of conductive particles, compared to the polymer binder, can eventually lead to a lack of structural integrity [28] (pp. 178–179). In this study, the conductive ink contains silver microparticles in the form of flakes with an average size of 1.2 µm.

UV inks that were used to formulate the insulating layers, polymerize only under exposure to UV light. To avoid the interference between the ink pigments and the polymerization reaction, we adopted a transparent UV ink type without pigments. A small amount of thinner ink was added to the UV ink to improve its transfer from the cliché to the substrate. UV inks formulation and polymerization are detailed in [29] (p. 222). The wavelength and the intensity of UV rays play an important role in the efficiency and the speed of polymerization as well as on the penetration depth of UV rays in the printed layers [30] (pp. 21–32). The effect of the intensity of the UV radiation on the polymerization of a layer of UV ink printed by inkjet on a silver-based layer was shown in [31], where solidification of the UV printed layer was achieved by using the minimum energy required. In this study, the required power for the polymerization of the UV ink is between 500 and 1000 mJ/cm^2^. To achieve homogeneity of the ink preparation, a mixing step is required. For this purpose, we used a mixing apparatus (SpeedMixer™ from Hauschild GmbH & Co. KG, Hamm, Germany) at 1300 rpm for 3 min.

The surface tension of the ink should be lower than that of the substrate to ensure appropriate transfer. In pad-printing, a value of at least 38 mN/m [32] (p. 70) is required for the ink to adhere well to the substrate’s surface. Depending on the intended application, the choice of the substrate must be well considered, since this choice is not always obvious and must be compatible with the printing process, its material, and its parameters. An appropriate transfer was obtained with substrates made out of Kapton^®^, paper, or wood [33].

Pad-printing pads play a determinant role in achieving satisfactory print quality. They are characterized by their surface conditions, their hardness, their size, their surface tension, and their geometries. Soft pads follow uneven surfaces better, however, with hard ones, the quality of patterns obtained is better. Having a size slightly larger than the size of the pattern and an adapted conicity to expel air bubbles without causing distortion, are the guidelines that we followed for the choice of pads.

Similarly, to other printing techniques, viscosity is one of the most important parameters in pad-printing since it plays a dual role, both in the ink transfer and the quality of each deposited film. Thus, appropriate viscosity allows correct cohesion of the ink as well as its adhesion on different surfaces. Viscosity changes over a transfer cycle time depending on the ambient temperature and the proportion of volatile components contained in the ink. Achieving an optimal printing quality demands the adaptation of ink transfer cycle times and speeds to the change of the viscosity of the inks during the printing process. Typical viscosity values of pad-printing inks vary between 1500 mPa∙s and 2000 mPa∙s [29] (p. 95). In this study, the viscosity of the conductive inks was 1897.5 ± 43.5 mPa∙s, while the viscosity of the UV insulating inks was 1485.9 ± 28.9 mPa∙s. These values were measured with a viscometer DV2T from BROOKFIELD.

Even though pad-printing does not require a cleanroom, a controlled working environment is necessary to achieve appropriate and repeatable printing qualities. The temperature and the humidity must be kept as constant as possible. High humidity affects, on the one hand, the evaporation of solvents in the inks and on the other hand the adhesion between the printed layer and the substrate as well as between the layers. The temperature plays an important role in the evaporation of the thinners and therefore in the ink transfer. Moreover, uncleaned surfaces introduce particles in the prints, which causes local smudge patterns and short circuits between adjacent tracks or between superimposed layers. Hence, regular cleanliness of the surfaces of substrates, pads and the engraved plate is important to achieving high-quality prints. The next step after cleaning and preparing the equipment is the configuration of the parameters of the pad-printing machine adapted to all the predetermined choices upstream.

A further recommendation is the application of an ionized airflow, which allows the neutralization of the free electric charge carriers and further removal of other particles from the printed surface. With the optimization of the printing velocity and the pad pressures on the cliché and the substrate, the edges were printed without smudges and filaments. Before the printing of the first layer, activation of the surfaces of pads and substrates was performed with isopropanol to increase the wettability of the ink deposition [34].

After each print of a conductive layer, an annealing process took place to achieve the curing of the formulated tracks. A “SalvisLab Thermocenter” (Renggli AG, Rotkreuz, Switzerland) was used for the annealing process of the conductive inks. While for the curing of the UV inks, a “UVP UVGL-58” handheld UV lamp (Analytik Jena AG, Jena, Germany), with 6 Watt power and two wavelengths of 254 nm and 365 nm, was used for their polymerization.

#### 2.2.2. Characterization Device

**Figure 2:**
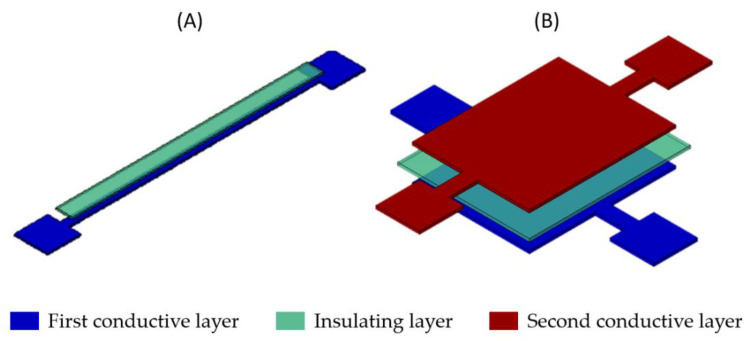
Schematic 3D view of two designs: (**A**) For the characterization of the ink conductivity and the tests of bending radius (**B**) For the characterization of the dielectric constant of the UV insulating ink.

Figure 2 depicts a 3D schematic view of two designs developed to evaluate the effectiveness and to characterize the resistivities of the conductive inks as well as the dielectric constant of the UV insulating ink. The first design (A) is conceived to check the printability of a conductive layer along a 1 mm × 30 mm thin line as well as to measure the resistance of this line. The same pattern has been used for the evaluation of the adhesion between the first layers and the substrate, the adhesion between layers, and the tests of the bending radius. The second design (B) is used to check the printability of three conductive-insulating-conductive layers on a 10 mm × 8 mm area as well as to test the insulation by measuring the capacitance between the two conductive layers as well as its breakdown voltage. Due to its smooth surface and thermal stability over a wide temperature range, Kapton was chosen as the substrate, not only because of its mechanical flexibility but also for its material stability allowing good printing quality.

Depending on the printed layer and the ink used, the machine parameters were adjusted in speed, pressure, and position before each printed layer. Each printing cycle had a duration of 7 s; this provides a printing speed of more than 500 prints per hour. The first conductive layer was pad-printed with three printing cycles and the samples were annealed afterwards at 100 °C for 12 h. The maximum temperature of the fabrication process in this study was arbitrarily set to 100 °C to maintain a low-temperature fabrication process and to prevent possible deterioration of the various materials involved. Next comes the printing of the UV insulating layer with three printing cycles as well. Exposure to UV rays and a waiting time of 15 min between the printing cycles allowed the crosslinking of the monomer network and the evaporation of the thinner.

Exposure in a dark room is also necessary to avoid interference with other wavelengths. Based on the intensity of the UV lamp, the requirements of the polymerization of the UV ink as well as the size of the printed samples, the minimal exposure time was estimated at 17 s. Therefore, the samples were exposed for at least 1 min at a distance of 10 cm from the UV lamp to ensure complete and thorough polymerization. Finally, the third conductive layer is printed with three printing cycles and then annealed at 100 °C for 12 h.

The adhesion of the layers to the substrate and the adhesion between layers has been evaluated according to the ASTM F1842-15 procedure, using a 3M^®^ 600 tape with 3.5 N/cm of 180° peel adhesion. The bending radius tests were performed with several 3D printed setups that allowed a deformation of 60 mm, 50 mm, 40 mm, 30 mm and 25 mm of bending curvatures.

An Optical contact angle measurement system “OCA200” (DataPhysics Instruments GmbH, Filderstadt, Germany) was used to measure the surface tension of each substrate.

A stylus profilometer “DektakXT” (Bruker AG, Billerica, MA, USA) was used to measure the thickness of each printed layer. An atomic force microscopy (AFM) “XE-100” (Park Systems Corp., Suwon, South Korea) was used to measure the roughness of the printed layers. An “1160 SERIES PROBE STATION” (Lucas Signatone Corp., Gilroy, California) was used to carry out four-point resistance measurements for the characterization of the conductive ink resistivity. The electrical measurements of resistances and capacitances were carried out using the “KEITHLEY DMM6500 6.5 DIGIT” multimeter (Tektronix^®^, Beaverton, OR, USA). A DC power supply “EX354RT” (Aim and Thurlby Thandar Instruments, Cambridgeshire, UK) was used for the evaluation of the breakdown voltage of the fabricated test devices.

#### 2.2.3. Demonstrator Fabrication

To verify the good functionality of this newly developed multilayer fabrication process, a simple electronic test circuit has been designed with three superimposed functional layers (conductive-insulating-conductive). This demonstrator is composed of SMD components typical for an IoT product. The demonstrator fulfils the function of controlling the lighting of certain LEDs arranged in a circle through the manipulation of a two-key capacitive touch sensor.

**Figure 3:**
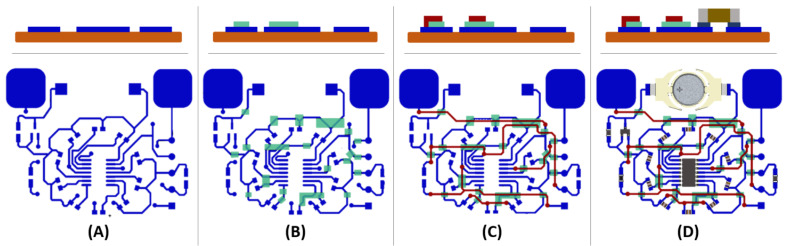
Schematic side views (**top**) and schema top views (**bottom**) of the multilayer demonstrator in the following fabrication steps: (**A**) Printing of the first conductive layer (in blue) with three printing cycles followed by annealing during 4 h at 100 °C. (The substrate is illustrated in orange in the side views). (**B**) Printing of the UV insulating layer (in green) with three printing cycles followed by a waiting time of 15 min, then exposure to UV radiation for 1 min. (**C**) Printing of the second conductive layer (in red) with three printing cycles followed by annealing during 4 h at 100 °C. (**D**) Mounting of electronic components where each electronic component has been fixed on its footprint by means of a conductive ink dispenser. This step is followed by annealing for 4 h at 100 °C.

The design and the necessary fabrication steps are detailed in Figure 3.

The software Altium^®^ was used for the schematic design and routing of the demonstrator circuit. The design has been imported to Adobe Illustrator^®^ to produce a suitable format for the production of the engraved plate. The software “MPLAB X^®^” was used to edit and compile the code in C language. A “PICkit™ 3” in-Circuit Debugger was used to compile the code into the microcontroller’s memory.

**Figure 4:**
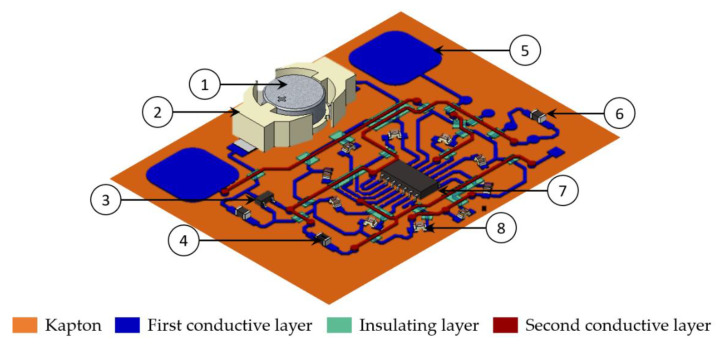
3D schematic view of the demonstrator composed of: (1) Battery, (2) Battery holder, (3) Voltage regulator, (4) Two 1 µF capacitors, (5) Two printed capacitive pads, (6) 10 kΩ resistor, (7) Microcontroller, and (8) Ten LEDs.

Figure 4 shows a 3D schematic view of the demonstrator. Besides the conductive and insulating tracks, the demonstrator circuit comprises: (1) One CR1225, 3 V, 50 mA battery [35], (2) One “SMTU1225-LF” battery holder [36], (3) One “MCP1700” LDO voltage regulator [37], (4) Two 1 µF capacitors [38], (5) Two printed capacitive pads, (6) One 10 kΩ resistor [39], (7) One “PIC16F1825” microcontroller [40], (8) Ten “SML-211UTT86” LEDs [41].

## 3. Results and Discussion

Open circuits and short circuits are the two main issues that need to be overcome when printing multilayers. The inhomogeneity of the deposited layer is an important source of these issues because areas with a lower thickness of the insulating printed layer will lead to a greater likelihood of breaking down with the applied voltage. In addition, cracks may appear due to the shrinkage of the insulation layer during annealing or due to external mechanical damage. Irregular lines and thin filaments may also appear at the edges of the printed films. These defects could create a short circuit and possible dysfunction of the printed components. The origins of these phenomena could be the formation of static electric charges on the pad and the result of an ink splitting effect that is correlated to the lift-off velocity of the pad from the cliché. An explanation of this issue and the optimization of the pad-printing parameters to improve the quality of the printed edges has been demonstrated in [42]. Trapped air bubbles that break, or rough surfaces (cliché, pad, substrate) could cause pinholes through the thickness of the film. The major problem we encountered when using traditional thinner-based inks, was the ineffectiveness of insulating layers printed on conductive layers. At the functional level, this inefficiency is due, on the one hand to the dissolution of the thinner of the newly printed insulating layer in the conductive layer located below, and on the other hand to the formation of air bubbles in the insulating layer that persists despite the increase in the number of printed layers.

**Figure 5:**
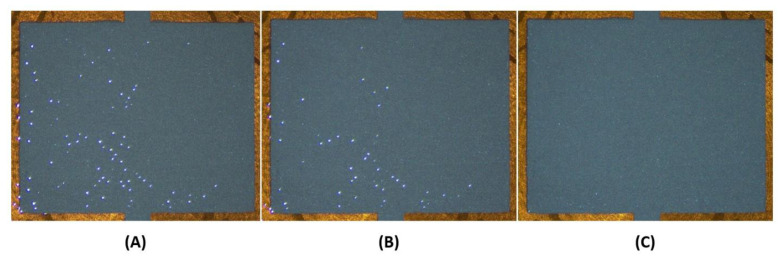
Photographic views of an insulating transparent UV layer printed on a silver-based layer: (**A**) 5 s after printing; (**B**) 10 s after printing; (**C**) 15 s after printing. These three views demonstrate the effect of the inks crosslinking, which leads to the disappearance of the pinholes over time.

A very interesting property of UV inks was observed within a minute after the pad-printing of this layer. This property is characterized by the crosslinking of the monomer matrices of UV inks, which is manifested by the closing of pinholes in the seconds following printing. After a few seconds, all the pinholes fade, and a homogeneous UV layer is formed as shown in Figure 5.

**Figure 6:**
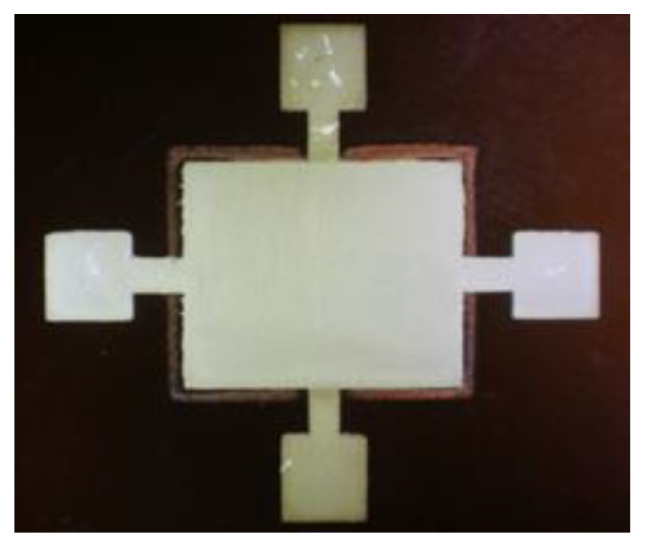
Photographic view of the multilayer test device printed on Kapton, which allowed the characterization of the dielectric constant of the UV insulating ink and the breakdown voltage.

The multilayer test device was successfully printed on Kapton as shown in Figure 6.

The average thickness of the conductive layers on Kapton is 19.5 ± 0.9 µm. The average thickness of the insulating layers printed on top of the first conductive layer is 8.4 ± 1.2 µm. The thickness of the ink film, deposited on the substrate in a single printing cycle, is significantly lower than the engraved pattern depth in the cliché. This difference is explained by three different effects. First, the transfer of the ink from the engraved plate to the pad is not complete and part of the ink remains in the engraved pattern. Second, the ink transfer from the pad to the substrate is not perfect either, leaving some ink on the pad. Last, an amount of the thinner is evaporated during printing and after drying.

Four-point measurements of the electrical resistance allowed us to estimate the resistivity of the conductive inks. The average measured resistance is 4.2 ± 0.2 Ω. Therefore, the resistance per square R□ is calculated at 1.4 Ω/□ and resistivity ρ is calculated at 2.73 × 10^−6^ Ωm. The relatively high resistance values, obtained in this study, might be improved by annealing at higher temperatures for a limited time as advised in [43,44]. It is important to emphasize that post-processing thermal annealing with high temperature and long duration mismatches with thermally sensitive substrates and rapid processing.

To evaluate the effectiveness of the pad-printing method in terms of layer adhesion, bending radius and fatigue resistance, several tests have been performed. According to ASTMF1842-15, The adhesion test results were rated to grade 4, meaning that limited delamination has been observed, which was less than 5%. The bending radius and fatigue resistance tests were performed for 10 samples, where both sides were deformed (convex for traction and concave for compression) for 100 cycles. None of the devices showed any mechanical fatigue traces on the printed layers. After performing the bending tests, less than 10% (0.40 ± 0.17 Ohm) average change in the absolute value of the resistance has been measured for the worst case (at 25 mm curvature).

**Figure 7:**
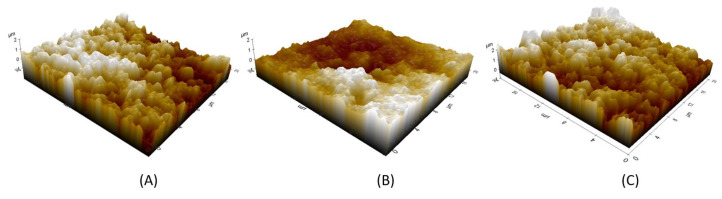
3D AFM images of the following superimposed printed layers on a 20 µm × 20 µm square: (**A**) First layer: conductive; (**B**) Second layer: insulating; (**C**) Third layer: conductive. Z range is set from −1 µm to 2 µm.

AFM measurements were performed on five printed samples of the multilayer test device (Figure 6 to analyze areas on an arbitrary 20 µm × 20 µm area as shown in Figure 7. The average roughness of the printed layers of the first conductive layer, the second insulating layer (on top of the first conductive layer), and the third conductive layer (on top of the two previous layers) is 969 ± 75 nm, 850.4 ± 113 nm, and 1249 ± 116 nm respectively. The high roughness of the surface of the conductive layer is mainly due to the presence of silver microparticles contained in the conductive ink. Smoother surface morphology is observed for the insulating layer which is deposited on top of the conductive layer. Hence, the insulating layer smoothens the roughness of the previously deposited layer and does not transfer its morphology entirely. In general, the morphology of each printed layer depends on the dispersion of the inks and their levelling properties. The roughness of the first conductive layer directly influences the needed thickness of the insulating layer. Reduction of the roughness of this first conductive layer would allow a thinner layer of the insulator and reduce the risk of its delamination.

The surface tensions measured for Kapton, and wood substrates were 56.9 ± 4.5 mN/m and 116.6 ± 17.1 mN/m respectively. These values explain the good printability of pad-printing on these types of substrates and the high wettability of wooden substrates. Surface tension measurements on paper were non-repetitive due to absorption.

The capacitance measurements carried out on five samples gave an average value of 281.8 ± 45.9 pF. This corresponds to a value of 3.5 pF/mm^2^. Thus, the calculated relative dielectric constant of UV ink is 3.3. The average breakdown voltage was measured at 38.8 ± 3.2 V. This value is suitable for most applications that can be found in printed circuits. In addition, the high roughness of the conductive layers below and above the insulating layer and its associated variations tends to limit the breakdown voltage. A post-process to smoothen the surface is expected to improve such a limitation.

Several aspects give UV inks advantages over conventional printing inks. The highly reduced amount of volatile organic compounds in the form of thinners in UV inks compared to thinner-based inks makes UV inks less harmful to operators and decreases materials used, thereby lowering the costs as well as the environmental impact of the manufacturing process [45,46].

This last advantage is accentuated by the fact that UV inks do not require curing methods based on heat, which are energy- and time-consuming processes; a fact that is translated into increased productivity and savings in energy resources thanks to their rapid polymerization (in this case, 6 watt UV lamp during 17 s). On an industrial scale, these two features could contribute to the reduction of the impact on the environment in terms of energy consumption, waste, and toxic substances released into the water and the air.

**Figure 8:**
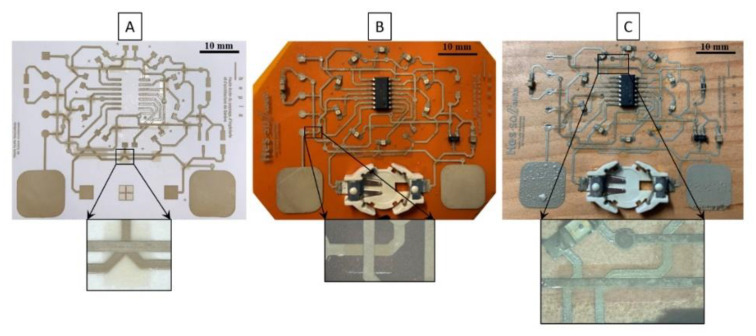
Photographic views of a multilayer demonstrator pad-printed on: (**A**) Cardboard, (**B**) Kapton, (**C**) Wooden board.

**Figure 9:**
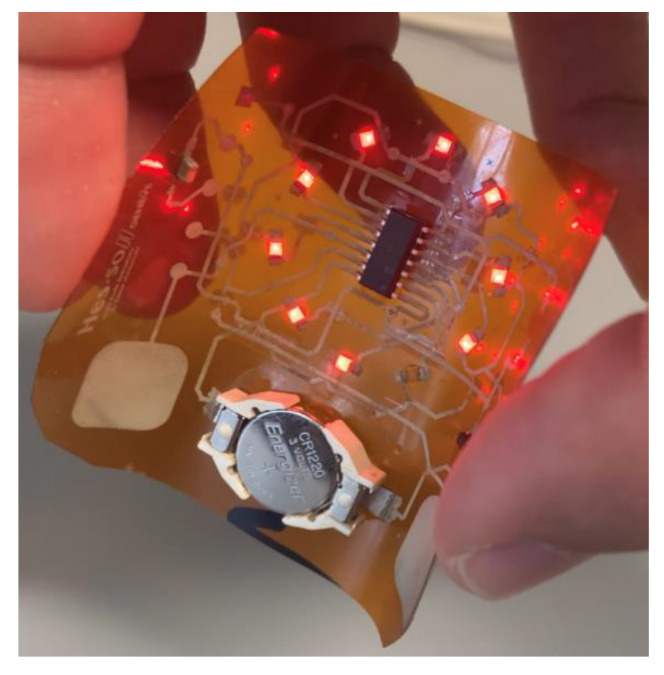
Photographic views of the demonstrator in operation. It was pad-printed on 127 µm thick flexible Kapton with three superimposed layers: first silver-based conductive layer, second UV insulating layer, and third silver-based conductive layer. The electronic circuit of the demonstrator controls the LEDs lighting by manipulating a two-key capacitive touch sensor.

Figure 8 presents the demonstrator’s multiple layers printed on three different substrates with specific zoomed areas of the superposition of the three layers. A correct printability of the UV layer is observed between the two conductive layers on the different substrates. Figure 9 depicts a photographic view of the demonstrator printed on Kapton as an industrial grade flexible circuit.

## 4. Conclusions

In this study, a process for the fabrication of multilayer circuits combining silver-based conductive and resin-based UV insulating layers has been successfully developed and tested. It is based on pad-printing, a 2D additive manufacturing process compatible with thin and flexible electronic circuits as well as conventional or non-conventional substrate materials. We established reliable recommendations to adjust the various parameters of this technique determining the quality of the obtained prints. Hence, a multilayer test device and a demonstrator circuit were successfully printed and analyzed. Both are composed of two conductive silver-based ink layers separated by one insulating UV ink layer.

An optimized fabrication method has been presented. It allows for high quality prints by overcoming inherent defects of pad-printing such as pinholes, layer misalignment, layer inhomogeneities, cracks, etc. The ability of UV inks to auto-repair and to form a homogeneous layer makes them ideal for the creation of insulating layers. In addition, their rapid polymerization highlights their superiority over traditional solvent-based inks, in terms of productivity, functionality, as well as environmental-friendliness.

To the best of our knowledge, this study is the first to demonstrate the potential of pad-printing as a process to create multilayer circuits. There are still limitations that deserve further exploration towards the increase in performance, simplicity, and robustness to reach an industrial grade of pad-printed multilayer circuits. The development of pad-printing conductive layers based on UV inks is one potential improvement that needs to be explored in the near future. This could potentially reduce fabrication cycle times and the need for solvents.

The particularity of pad-printing combined with the attractive properties of UV ink presages the potential of this approach to arouse the interest in a wide range of applications in printed and wearable electronics.

## Data Availability

The data presented in this study are available on request from the corresponding author.
